# The Generation of an ocular anti-inflammatory biotherapeutic to enhance wound healing.

**DOI:** 10.21203/rs.3.rs-4354377/v1

**Published:** 2024-08-16

**Authors:** Anthony St. Leger, Jackie Shane, Matthew Evans, Yannis Rigas, Robert Shanks

## Abstract

Microbes exist at and colonize mucosal surfaces striking a balance with the host immune system, so that these microbes can thrive on host tissues without causing pathology. Because of this, mucosal barrier-colonizing bacteria can be leveraged to act as long-term delivery vehicles for naturally derived therapeutics. Here, we use a mouse model of corneal wound healing to show that the eye-colonizing bacterium, Corynebacterium mastitidis (C. mast) can be engineered to produce and secrete bioactive murine anti-inflammatory interleukin (mIL)-10. Specifically, we used transposon mutagenesis to identify a native C. mast-specific secretion signal that was used to direct C. mast to secrete mIL-10. Mini-transposons were generated to deliver secretion capable mIL-10 to the bacterial genome. After screening, two isolates were identified that can: 1) colonize the eye, 2) produce and secrete mIL-10, and 3) enhance wound healing in an IL-10-dependent manner. This proof of concept illustrates that eye-colonizing bacteria can be engineered to deliver therapeutics to the ocular surface for the alleviation of ocular surface disease(s).

## Introduction

Corneal wounds remain a serious concern for ocular surface health as over 1 million Americans are afflicted with corneal wounds annually and they account for 3% of all visits to the emergency room [1]. During wound healing, the eye is susceptible to infection and inflammatory damage, which can lead to other more serious issues like nerve damage[2,3], opacification, and loss of visual acuity[4,5]. Therefore, increasing the rate of wound healing is paramount in preventing long-term disease.

Corneal wound healing relies on an appropriately mounted immune response that encourages healing and limits inflammation. After corneal wound healing, the upregulation of inflammatory cytokines, interleukin (IL)-1b, IL-6, and TNFa, suppresses STAT3 activity, which can impair the proliferation of corneal epithelium and disrupts normal healing of the cornea[6,7]. Alternatively, IL-17 from cornea-infiltrating ɣδ T cells can activate STAT3, which enhances wound healing[8,9]. Similarly, IL-10 can also activate STAT3 to encourage proliferation and migration of epithelial cells both in the cornea[7] and other barrier surfaces throughout the body[7,10,11]. IL-10 also directly inhibit inflammation by suppressing cytokine production and proliferation of inflammatory myeloid cells. Furthermore, mIL-10 directly interfere with the activation of T cells that require CD28 co-stimulation[12-14] and suppresses T cells that exacerbate inflammation in many ocular surface diseases like infectious keratitis and Dry Eye Disease [2,15,16]

Virtually all barrier sites have a resident microbiome that directly interacts with the host and fosters tissue barrier function[17]. Microbial members of the microbiome have adapted mechanisms that allow them to thrive at the sites of colonization. These unique adaptations make them attractive candidates to continually deliver naturally derived therapeutics to sites of interest. Indeed, scientists have begun engineering components of the intestinal microbiome to modulate metabolism[18] as well as cytokine production to regulate immunopathology associated with mouse models of colitis [19-21] and clinical trials as a therapeutic for Crohn's disease[22]. When IL-10 was expressed by *Lactococcus lactis*, a gut bacterium, CD4+ T cell-mediated colitis was reduced[21]; however, the direct effects of mIL-10 signaling in this model are still unclear. On the ocular surface, therapeutics are continually washed away or diluted by the flow of tears[4,23], so engineering eye-colonizing bacteria that can deliver therapeutics to the ocular surface is a viable avenue towards reducing the burden of applying therapies like eye drops multiple times per day.

Recently attention has been directed to host-microbiome interactions at low biomass sites like the lungs, skin, and eye and how those microbes may modulate physiology. Identifying relevant and tissue-colonizing bacteria has been difficult at these sites due to their paucibacterial nature; however, an eye-colonizing bacterium, *Corynebacterium mastitidis* (*C. mast*), has been isolated and cultured from the mouse conjunctiva. While *C. mast* elicits immune responses at the ocular surface, colonization of immunocompetent hosts does not result in excessive inflammation or pathology. Therefore, *C. mast* is an attractive target for engineering as it appears non-pathogenic during homeostasis[24].

Many factors must be considered in the development of an ocular live biotherapeutic product (LBP). With a few exceptions, like *Bacillus subtilis*, Gram-positive bacteria can be very difficult to manipulate genetically. Until recently, there was no established set of tools for expressing or manipulating genes in *C.mast*. In this study, we employed a method of transposon mutagenesis, which has previously resulted in successful genetic manipulation of *C. mast*[25] . Another consideration for the development of LBP is that the therapeutic of interest must be secreted from the bacterium, so that it can interact with surrounding host cells. Therefore, a secretion signal that is unique for the selected bacterium—*C. mast*—is essential for proper development of a physiologically relevant ocular LBP. A final consideration for these studies is retaining the ability to colonize the ocular surface after genetic manipulation. This allows for the continual delivery of the therapeutic to the tissue of interest. Given the beneficial immune regulatory and tissue repair characteristics following IL-10 signaling, we engineered *C. mast* to produce and secrete mIL-10 while maintaining the machinery required to colonize the eye.

In this study, we have identified a novel secretion signal from *C. mast* using a phosphatase transposon mutant library. After incorporating the secretion signal upstream of the gene for mIL-10 into a transposon cassette, we introduced the gene cassette into the *C. mast* genome by transposition. Multiple candidates produced and secreted mIL-10 *in vitro* and *in vivo*, which led to enhanced wound healing when mIL-10-*C. mast* colonized the eye. Here, we reveal the first proof-of-concept that an ocular bacterium can be genetically engineered to be an LBP to speed corneal wound healing. Our results also suggest that this technology can be expanded to include other therapeutics that may be delivered to the ocular surface to treat disease.

## Methods

#### Bacterial culture and genetic constructs

*C. mastitidis* AS1was isolated from ocular swabs and cultured with LB media containing 0.5% Tween-80 grown aerobically at 37°C. For T-cell culture experiments cell culture media was used consisting of DMEM with 10% FBS; 1% NEAA, glutamax, Hepes, and sodium pyruvate. Bacteria added to T-cell culture used the same cell culture media with the addition 0.5% corn oil and 0.05% w/v casamino acids. The phosphatase Z gene was isolated via PCR from DNA extracted from *Enterococcus faecalis* using primers indicated in Table 1. The *AphA3* kanamycin resistance gene construct was isolated froma previously created plasmid, pTony3[25], and the erythromycin resistance gene cassette was created by using the same promoter attached to a codon harmonized erythromycin resistance gene of *C. striatum*. Codon harmonized mIL-10 was ordered as a gBlock Gene Fragment (IDT, USA). Codon harmonization was performed using the online Galaxy software, Codon Harmonizer[26]. All newly created sequences can be found at accession # PP471979- PP471980 and all primers used are detailed in Table 1.

#### Mice

Female C57BL/6 mice purchased from Jackson Laboratories (Bar Harbor, ME, USA) were housed in the Animal Resource Facility at the University of Pittsburgh Medical Center (Pittsburgh, PA, USA) and colonized with *C. mast* at 6 weeks of age in all experiments according to our previous inoculation schemes. Briefly, mice were ocularly inoculated with $1x10^6$ CFU of *C. mast* every other day for a total of 3 inoculations. Two weeks after the final inoculation, the corneas were wounded as described below. Experimental procedures were reviewed and approved by the University of Pittsburgh Institutional Animal Care and Use Committee and the use of animals was in accordance with the ARVO Statement for the Use of Animals in Ophthalmic and Vision Research.

**Generation of the secretion cassette.**

Secretion signals for *C. mast* were identified by creating a phosphatase (PhoZ) transposon library. Using Overlap extension PCR (OEP), promoterless *phoZ* lacking its secretion signal from *Enterococcus faecalis* was fused with an *AphA3* kanamycin resistance gene from a previously created plasmid, pTony3[25],to create a transposome that was inserted randomly into the wild-type *C.mast* genome using EZ-Tn5™(Lucigen). Positive mutants secreting PhoZ were selected for on kanamycin plates (100 µg/ml) supplemented with BCIP (5-bromo-4-chloro-3'-indolyphosphate p-toluidine salt) (25µg/ml) that turns colonies blue when phosphatase is secreted. Blue colonies were grown in broth to log phase and supernatant was collected and analyzed using the 1-Step™ PNPP Substrate Solution from ThermoFisher and repeated in triplicate. The genomes from the best secreting isolates were sequenced via Illumina whole genome sequencing,150 Mbp: 66 times genome coverage, to find the insertion site of the transposome cassette. The insertion site sequence was analyzed using signalP 5.0[27] to find the strongest predicted secretion signal.

**mIL-10 production and functionality in-vitro**

The DNA sequence of the strongest signal was added to a codon harmonized mouse-IL-10 gene preceded by a strong constitutive promoter in a synthesized double stranded DNA fragment (gBlock™ Gene Fragment, IDT, USA). This was fused with a harmonized erythromycin resistance gene from *C. striatum* using overlap extension PCR to create the new transposome cassette and inserted into the *C.mast* genome. Positive isolates were screened using antibiotic selection (100 µg/ml) with over 200 positive mutants collected. These were grown to log phase and screened for mil-10 production in their supernatant using IL-10 Mouse Uncoated ELISA kit (ThermoFisher) and normalized to $OD_{600}$. In three separate experiments, the best cytokine secreting isolates were verified by growing each to exactly 0.5 $OD_{600}$ and quantifying supernatant mIL-10. The functionality of the cytokine was tested *in vitro* by analyzing the effect of mutant supernatant on t-cell proliferation. Naïve ($CD44^-CD62L^+$) or memory ($CD44^{hi}CD62L^{low}$) $CD8^+$, $CD4^+$ and ɣδ T cells were isolated by fluorescence activated cell sorting (FACS) on a Sony Biotechnology MA900 cell sorter. After sorting, cells were labeled with a proliferation dye (Cell Trace™ Invitrogen) and stimulated for 3 days with plate-bound aCD3 (1mg/ml) and soluble aCD28 (5mg/ml)l. Six hours after the initial stimulation, 10 µl of supernatant from log-phase bacterial cell culture broth of AS1 or mIL-10 secreting mutants. After stimulation, proliferation was measured via flow cytometry using a Beckman Coulter Cytoflex LX. T-cell culture supernatants from these assays were then assessed for IFN-γ and IL-17 production using ELISA (BioLegend).

**Fitness of mil-10 transposon mutants**

To assess the fitness of the mIL-10 secreting mutants they competed with wild-type (WT) *C. mast* in the same broth culture through three separate experiments. Transposon mutants and WT AS1 were grown to log phase in liquid media, pelleted, washed and resuspended at 0.5 $OD_{600}$ in media without antibiotic. 1mL from each transposon mutant resuspension was mixed with 1 ml of WT and allowed to grow together for 6 hours. The cultures were then ten-fold serially diluted to $10^{-8}$ and 100ml was plated on agar both with and without antibiotic to select for the mutant. Comparing the CFU on each of the plates allows for the quantification of the ratio of mutant bacteria contained in the culture.

These isolates were also evaluated in-vivo for their ability to colonize the mouse eye. Transposon mutants and WT AS1 were grown to log phase in liquid media Then pelleted and resuspended at $1x10^6$ CFU/ml in PBS. Five mice per sample were inoculated with 5 µl of each mutant, control WT and PBS dropped onto mouse eyes that had been brushed with a sterile swab to disrupt the tear film. This was repeated every other day for a total of 3 inoculations. Two weeks after the last inoculation mouse eyes were swabbed and plated on selective media. Colonies were counted and compared to the colonization rate of WT AS1 to determine *in vivo* fitness in three separate experiments.

### In-vivo corneal wounds

To evaluate the effect of the mIL-10 producing mutants on the ocular surface we inoculated the eyes as previously described and bacteria were quantified to ensure colonization and no contamination. Two weeks after the last inoculation a 2.5mm epithelial corneal wound was made on the right eye of each mouse using an Alger brush without disrupting the basement membrane. A 0.01% Fluorescein solution was then dropped onto the eye and pictures were taken at T=0. Fluorescein-stained cornea pictures were again taken at T=10 hours and every three hours after until the first wounds showed full closure at 22 hours. The size of the wound at each time point without full closure was measured by increasing the saturation of only the green pigment in the image and using the analyze particles function of Fuji (ImageJ). The size at each timepoint was then compared to the size at T=0 to find the amount of healing at each time point for each individual mouse. These data were replicated with three total experiments.

Finally, the mIL-10R was blocked *in vivo* using CD210 antibody administration (Bio X Cell). Two weeks after the final bacterial inoculation, 200 mg of CD210 in 1X PBS was injected intraperitoneally (IP). Controls were given IP injections of IgG. Twenty-four hours after injection corneal wounds were performed and analyzed at t=0 and t=19 hours as previously described. The amount of healing for each isolate was compared between CD210 treated mice and IgG injected mice as well as each isolate compared to the WT-control.

### Statistics

All ANOVA and Student's t-tests were performed using Graphpad PRISM software. Comparisons across more than 2 samples used an ANOVA with Dunnett's multiple comparisons test to the WT AS1 sample. When only 2 samples were compared a student's t-test was performed. For the *in vitro* competition assay a one-tailed single sample t-test was performed with an expected mean of 0.5. When comparing epithelial wounds with and without IL-10R blocking an unpaired student's t-test was used. Only wounds that healed at least 15% after 19 hours were included in the analysis. All error bars represent standard error of the mean.

**Data Availability**

The transposon sequences are available in the Genbank database with accession numbers PP471979 and PP471980. Source data are provided with this paper.

## Results

**Identification of the novel *C. mast*-specific secretion signals.**

Bacteria use secretion signals to release proteins into the extracellular milieu. These secretion signals can be highly specialized and specific to the bacterium of interest[28]. Because *C. mast* secretion signals are currently unknown, we created a phosphatase (PhoZ) transposon *C. mast* library to identify *C. mast*-specific secretion signals. We used transposon mutagenesis to incorporate a gene cassette containing kanamycin resistance and the *phoZ* reporter gene into the *C. mast* genome. When grown on agar plates supplemented with 5-Bromo-4-chloro-3-indolyl phosphate (BCIP)—substrate for PhoZ—mutant colonies that actively secrete PhoZ appear blue because BCIP cannot penetrate the cell membrane. (Fig. 1a). Secretion of PhoZ by positive transformants was quantified by growing mutants in liquid media to log phase and analyzing phosphatase activity in the supernatant using a p-Nitrophenyl phosphate (PNPP) assay (Fig. 1b). DNA from the four isolates with the significantly greater concentration of secreted protein was purified for whole genome sequencing to identify the *C. mast* genes containing the transposon insert. We found that the isolate with highest secretion activity, isolate 21, had the transposon insert within an adhesin associated protein (accession# WP_157824337). Using the SignalP 5.0[27] online software we isolated the signal sequence and characterized it as a Sec/SpI; this signal peptide was used to make the mIL-10 transposon.

**mIL-10 produced by genetically engineered *C. mast* can suppress T cell activation *in vitro.***

After identifying the active *C. mast* secretion signal, we generated a new cassette that consisted of the adhesion secretion signal upstream of a codon harmonized mIL-10 and an antibiotic resistance gene. To incorporate this cassette into the *C. mast* genome, we generated another set of transposomes according to our previous study[25], which was used to generate another transposon mutant library. After screening supernatants of the transposon mutant library, we found over 50 isolates that secreted ELISA detectable mIL-10; these were collected, and the three best candidates are shown in Fig. 2a. These data suggested that *C.mast* properly produced and secreted mIL-10 that is recognized by commercially available antibodies. The transposons for these isolates were inserted into the genes for trehalose 6 phosphate, DnaK, and 23s rRNA for mil10.4,13, and 14 respectively.

Knowing that mIL-10 is glycosylated[29], we wanted to ensure that mIL-10 was bioactive in cells that would respond to IL-10 signaling. To address this, we isolated memory ($CD44^{hi}CD62L^{low}$) $CD4^+$, $CD8^+$, and ɣδ T cells from C57BL/6 mice. We non-specifically activated these cells by stimulating αβ T cells with plate-bound aCD3 and soluble aCD28 for 3 days. In the presence of supernatants from mIL-10-*C. mast*, T-cells proliferated to a lesser extent as compared to T cells stimulated in supernatants from WT *C. mast* (AS1) (Fig. 2b). Similarly, the production of the proinflammatory cytokine, IFNg, was inhibited by supernatants from IL-10-*C.* mast, which supports the regulatory nature of mIL-10 during *in vitro* stimulations demonstrating functionality (Fig. 2c). Notably, the cytokine, IL-17, which is required for optimal protection of the ocular surface from spontaneous infection[30], was not inhibited by IL-10-*C. mast* supernatants (Fig. 2d). Because IL-17 is not reduced in the presence of supernatants from IL-10-*C. mast*, we posit that IL-10-*C. mast* does not affect the host-microbe interactions concerning the ocular microbiome.

**IL-10-*C. mast* has a similar *in vivo* fitness compared to wild-type *C. mast***

To assess whether IL-10-*C. mast* would survive a physiological environment, we performed *in vitro* and *in vivo* experiments to quantify fitness. First, mutant and WT strains of *C.*

*mast* were grown to log phase and mixed at a 1:1 ratio. This allowed a direct comparison of growth between each mutant and WT *C. mast* (Fig. 3a) After 6 hours in liquid broth, isolates 4 and 14 did not have a significantly lower ratio of mutant bacteria as the initial time point. Conversely, isolate 13 trended towards a reduced ability to compete with WT *C. mast,* showing a significantly reduced ratio of mutant vs WT bacteria as in the initial culture. We next analyzed the mutants for their ability to colonize the mouse conjunctiva. Five million CFU of log-phase mutant or WT *C.mast* were applied to the eye every other day for a total of 3 inoculations. After 1 week, eyes were swabbed, and colonizing bacteria were quantified. Both isolate 4 and 14 were able to colonize the mouse eye with no significant change in CFU compared to WT while isolate 13 was unable to colonize the mouse eye at all (Fig 4b). These data indicate that neither isolate 4 nor 14 were significantly less fit than WT *C. mast*; however; isolate 13 was significantly impaired in its ability to colonize the eye. Given that isolate 13 could not colonize the eye, and that the transposon cassette was incorporated into the DnaK gene, we conclude that the transposon impaired the function of the critical DnaK chaperon protein and reduced the fitness of isolate 13 to the point that colonization was not possible.

**IL-10 produced by *C. mast* can speed wound healing in an IL-10 dependent manner.**

To assess the therapeutic potential of IL-10-*C. mast,* we asked whether the secreted mIL-10 could affect physiology at the ocular surface. To do this, we used a mouse model of corneal wound healing due to the advantageous nature that IL-10 has on the closing of corneal wounds. One week after mice were ocularly colonized with either WT *C. mast,* IL-10-*C. mast,* or PBS, a 2.5 mm abrasion of the corneal epithelium was made using an Alger brush. Fluorescein imaging was used to assess the size of the wounded area at the time of injury and until 19 hours after injury (Fig. 4a&b) The IL-10 secreting bacteria significantly increased the rate of wound healing compared to controls. To ensure that the observed effects were directly related to IL-10 signaling, we administered IL-10R blocking antibody (CD210) intraperitoneally 24 hours prior to wounding and we observed fluorescein staining at t=0 and t=19 hours (Fig. 4c). Our results showed that wound healing was significantly slowed after CD210 administration in mice colonized with IL-10-*C. mast.* CD210 treated mice that were colonized with IL-10-*C. mast* had the same rate of wound healing as PBS and WT AS1 controls supporting the notion that engineered IL-10 was biologically functional and was responsible for the increased rate of wound healing after corneal injury. Together, this outlines the therapeutic potential of engineering an ocular resident microbe to deliver bioactive therapeutics directly to the ocular surface to treat disease.

## Discussion

The ocular surface continues to be a difficult tissue for the application of therapeutics given the sensitive nature of the cornea, aversion of patients to touching the eye, and the constant flow of tears across the ocular surface. Current standards of care for local delivery of therapeutics include invasive and/or laborious methods that include subconjunctival injections and topical application up to once every 2 hours. To remedy this, we devised a strategy where we would engineer the known eye-colonizer, *C. mast*, to produce and secrete an anti-inflammatory and pro-wound healing cytokine. In this study, given the anti-inflammatory and pro-healing qualities of IL-10, we decided to engineer *C. mast* to secrete IL-10 at the ocular surface.

Despite *Corynebacterium spp.* having been engineered in the past, most studies have focused on engineering *C. glutamicum*—a soil-habiting bacterium—to produce amino acids in an industrial production setting[31] , our study; however, focused on genetically manipulating *C. mast* for the purposes of generating a therapeutic delivery vehicle or a live biotherapeutic product (LBP). The benefits LBPs are that colonization with the LBP negates the need for repeated treatment regimens that reduce compliance. Similarly, LBP therapies could theoretically reduce production costs as the therapeutic is derived from the bacterium rather than synthetic methods. Furthermore, LBPs have proven effective in pre-clinical and some clinical trials involving Crohn's disease and irritable bowel syndrome (IBS)[20,22] . More recently, IL-10 produced in *Mycoplasma pneumoniae,* was shown to be effective at limiting the inflammatory response related to *Pseudomonas aeruginosa* lung infection[32]. With that, we hypothesized that IL-10 delivered by an eye-colonizing microbe may be beneficial for ocular surface health. Indeed, the production and release of IL-10 from *C. mast* resulted in more efficient tissue repair at the ocular surface after corneal injury. This suggests that this may be an effective method of treatment for traumatic ocular surface injuries, which affect over 1 million Americans per year[1]. Conversely, how this system would affect infectious ocular surface disease has yet to be determined.

This strain of *C. mast* was originally isolated from ocular surface and skin of mice, but unlike laboratory strains of bacteria, genetic tools and protocols to manipulate *C. mast* have not been well-developed. Recently, our group used transposon mutagenesis to incorporate the gene of mCherry into the *C. mast* genome[25]. While we used this same system to incorporate IL-10 into *C. mast*, in this study, we needed to identify a signal sequence, which we could use to direct *C. mast* to secrete IL-10 into the extracellular space. Through the PhoZ transposon library we discovered a strong Sec/SPI signal peptide, a standard signal that is trafficked outside the cell by the Sec translocon and cleaved by Signal Peptidase I, that would serve this purpose.

A difficulty in modifying host immune responses is the balance of regulating inflammation, wound repair, and preventing infection. Given that we induced expression of IL-10, an immune regulatory cytokine, we were concerned that the continual production and release of IL-10 may affect the growth and control of *C. mast* resulting in *C. mast* becoming a pathobiont. Notably, we observed that IL-10 from *C. mast* reduced the inflammatory cytokine, IFNg, but it did not affect the production of IL-17 from ɣδ T cells, which is produced in response to *C. mast* colonization. More importantly, we monitored mice colonized with WT *C. mast* and IL-10-*C. mast* for multiple months (data not shown) and found that neither caused clinical signs of pathology during colonization. Similarly, there were no differences in the host response—IL-17 from ɣδ T cells or neutrophil recruitment—to colonization with either WT *C. mast* or IL-10-*C. mast*. Therefore, we concluded that even though IL-10-*C. mast* produced an immune regulating cytokine, it is unlikely that this genetically engineered microbe would become pathogenic in an immune competent host. Because of our engineered *C. mast*'s ability to remain long-term, induce IL-17 immunity similar to WT, and lack of pathology, we demonstrate that use of engineered *C. mast* as an LBP has the potential to be well-tolerated and beneficial in a clinical setting.

LBPs such as this still require an incredible amount of tuning. Here, we simplified the system to ensure that we reduced as many variables as possible. Indeed, we were able to focus our efforts on identifying a secretion signal and ensuring that functional IL-10 was produced during colonization. Future iterations of this technology may rely on other cytokines and/or factors that may better tune the ocular surface microenvironment to encourage homeostasis. Specifically, there may be proteins or cytokines that help reduce inflammation related to Sjogren's Syndrome and severe dry eye disease. Alternatively, it may be possible to use this technology to produce growth factors that may assist the longevity of adoptively transferred stem cells or reduce the possibility of corneal graft rejection. On the microbe side, since this bacterium is susceptible to a number of antibiotics used for the eye, it can be removed from the ocular surface if desired, and work to determine optimal approach is underway.

Most LBP research has focused on influencing the host-gut microbiome axis; however, more attention is being directed at low biomass sites. Recently, LBPs have proven useful in the lungs[32,33]; however here, we have demonstrated the first proof-of-concept of an LBP that can colonize the ocular surface. Specifically, the purpose of this LBP is to treat ocular surface wounds, but this technology can likely extend to other diseases at the ocular surface. In mice, this LBP was well-tolerated and did not result in uncontrolled outgrowth or gross pathology. Additionally, this LBP secreted physiological levels of functional IL-10 in culture and *in vivo* at the ocular surface, which demonstrates that this technology may negate the need for burdensome treatment regimens that need to be applied to the eye multiple times a day. In the future, using engineered ocular resident microbes as LBPs may provide to be an alternative approach to treat ocular surface diseases.

### Acknowledgements

The work was supported by NIH grants: R01EY032482 (AJS), U24EY035102 (AJS), T32EY017271 (JS), and P30EY008098. In addition, we acknowledge the support from the Hillman Innovation Exploratory Award, Eye & Ear Foundation of Pittsburgh, and unrestricted grants from Research to Prevent Blindness Inc, New York, NY. We would also like to thank Dr. Daria Van Tyne for generously providing *Enterococcus faecalis*, which was used to isolate the phosphatase Z gene.

### Author Contributions

Conceptual design of the study and method implementation: J.S., A.J.S.; *In vitro* stimulations and flow cytometry: Y.R., A.J.S.; Strategizing and performing genetic modification of *C. mast*: J.S. R.M.Q.S., M.E., A.J.S.; Writing of the manuscript: J.S., A.J.S.; Critical reviewing of the manuscript: all authors.

### Competing Interests

The authors have no financial or competing interests to declare.

### Figures

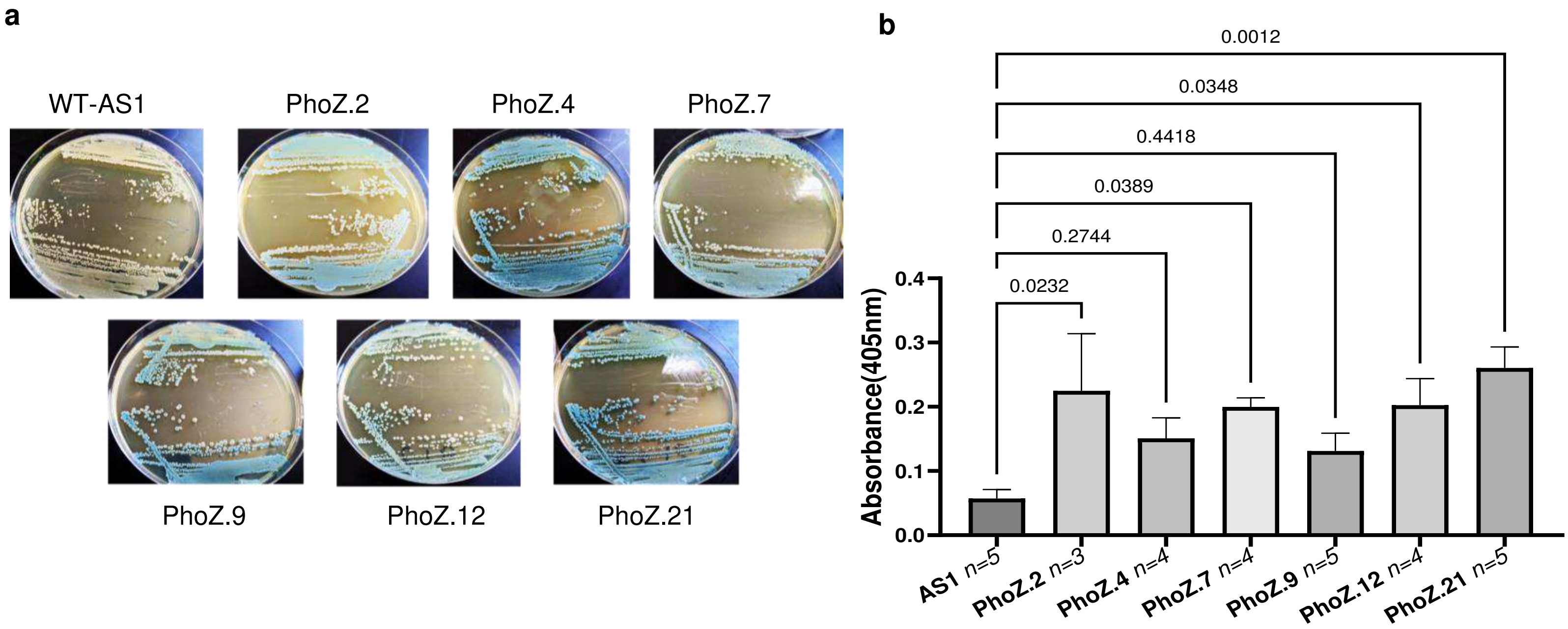

**Figure 1. Identification of the novel *C. mast*-specific secretion signals.** (a) Representative images of PhoZ secreting *C. mast* mutants created using EZ-TN5 transposase and a phosphatase/ kanamycin resistance transposon gene cassette. Six transposon mutants analyzed from the library were able to significantly secrete phosphatase outside the cell, demonstrated by colorimetric change on agar supplemented with 5-Bromo-4-chloro-3-indolyl phosphate (BCIP) repeated in triplicate. (b) Absorbances after p-Nitrophenyl Phosphate (PNPP) were quantified across all six isolates and WT control. Bars represent mean average absorbance ± SEM. Experiment was performed at least 3 independent times and averages of technical duplicates used for each isolate. Significance was determined using ANOVA (F=4.041, df= (6, 23)) and compared to the WT *C. mast* (AS1) control.

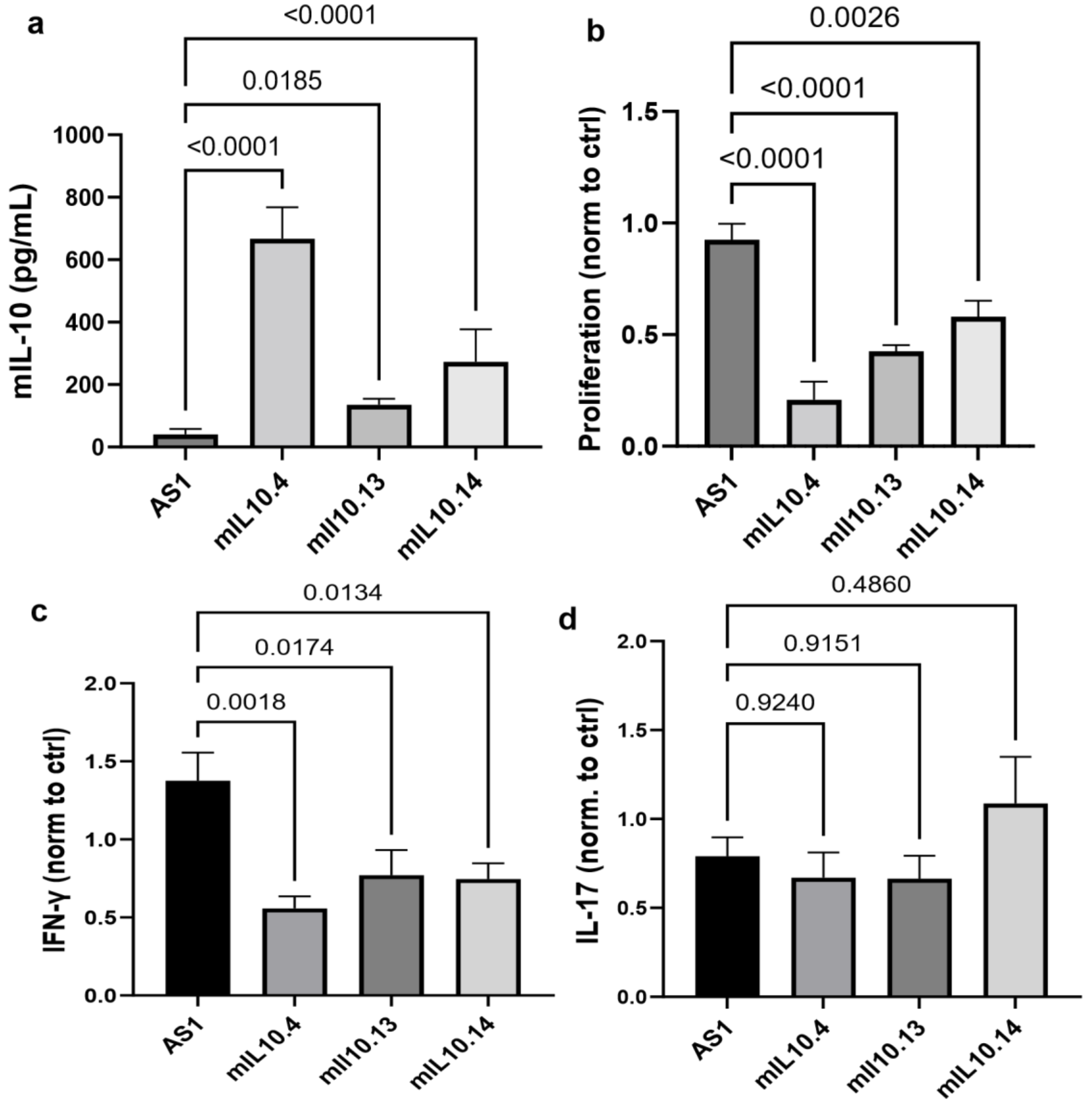

**Figure 2.** ***C. mast*** **mutants can secrete functional mIL-10 that induces immunological changes in T-cell culture.** (a) Two biological replicates of control and transposon mutant bacteria were grown to log phase and each culture was pelleted. Supernatants from bacteria cultures were assessed by ELISA. Concentrations were extrapolated from absorbances acquired from the IL-10 standard curve. Bars represent the mean IL-10 concentration ± SEM. Data were pooled from three independent experiments (n=6, F = 5.639, df= (6, 12)). (b-d) Memory ($CD44^{HI}CD62L^{LOW}$) T cells (αβ and ɣδ T cells) isolated by FACS, labeled with Cell Trace Violet Dye, and were stimulated with plate-bound aCD3 and soluble CD28 for 4 days. Bacteria were grown to log phase and supernatants from AS1 or the mIL10 transposon mutants was added to cultures after 24 hours. (b) Proliferation was measured by flow cytometry as determined by a loss of cell trace dye. Data were normalized to the media control. Bars represent the mean fold change in proliferation ± SEM. Data were pooled from three independent experiments (n=8, F=20.87, df=(3,28)). (c & d). Four days after the initiation of stimulation, brefeldin A was added to stimulated T cells and cytokines were assessed by flow cytometry. Data were normalized to the media control. Bars represent the mean fold change in (c) IFN-g or (d) IL-17 production ± SEM. Data were pooled from five independent experiments and data were averaged from three technical replicates. p values were determined using ANOVA (n=5, F=6.713; 1.350, df=(3,16)).

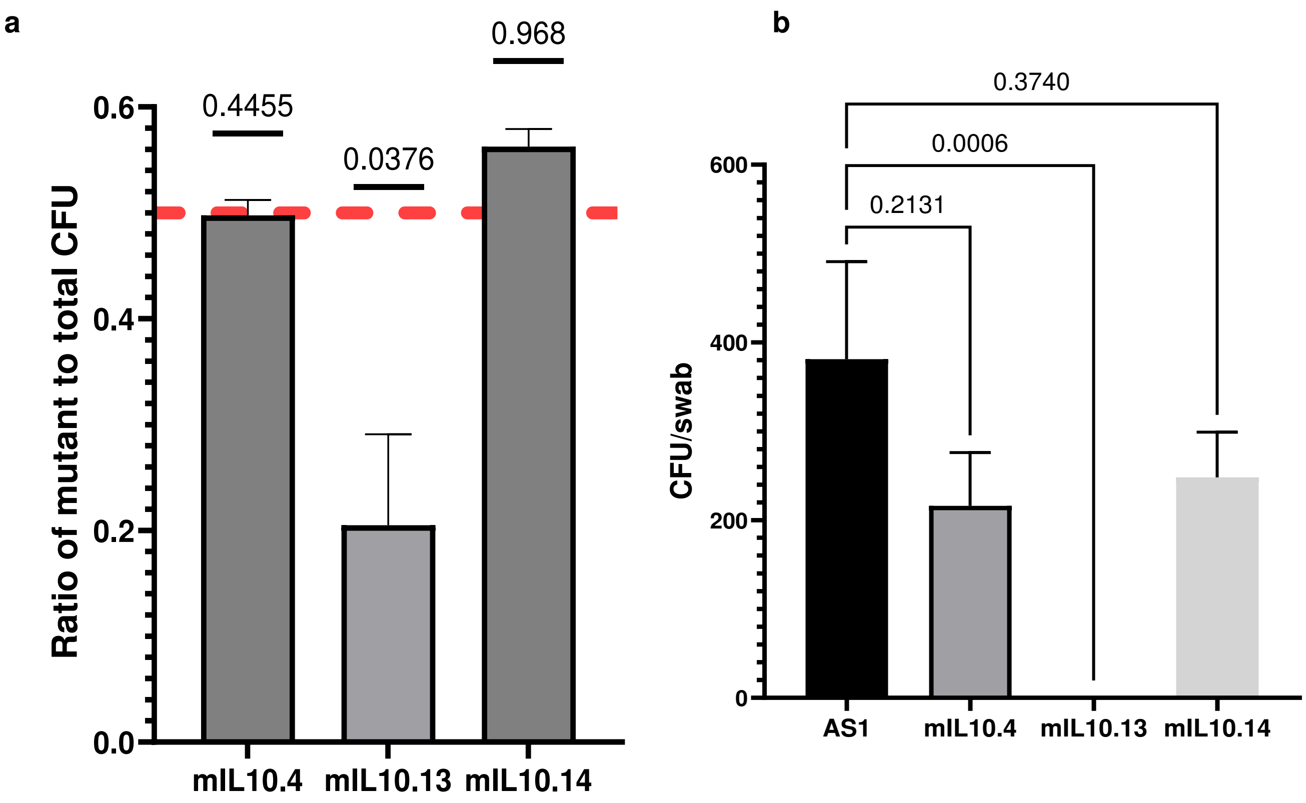

**Figure 3. IL-10-*C. mast* has a similar in vivo fitness compared to wild-type *C. mast*.** (a) Log phase mutant and WT bacteria were equally mixed in antibiotic free LB at 0.5 $OD_{600}$. They were grown for 6 hours before being plated on agar with and without antibiotic. Comparing the CFU on each plate the ratio of mutant vs WT AS1 was determined. Bars represent the mean ratio of mutant CFU to total CFU ± SEM. Data were pooled from three independent experiments(n=3) and p values calculated using one-tailed single sample t-test with an expected mean of 0.5(n=3 df=2; 4(t=-0.1552); 13(t=-3.4368); 14(t=3.7634)). (b) $5x10^6$ CFU of bacteria were applied to the ocular surface of C57BL/6 mice once every other day for a total of three inoculations. One week after the final inoculation, the eyes of mice were swabbed and CFU per swab were quantified using agar plates. Bars represent the mean CFU/eye ± SEM. Individual n values were from individual mice. Data were pooled from 3 experiments and statistical p values were determined using ANOVA (n=14, F=5.468, df=3,52)).

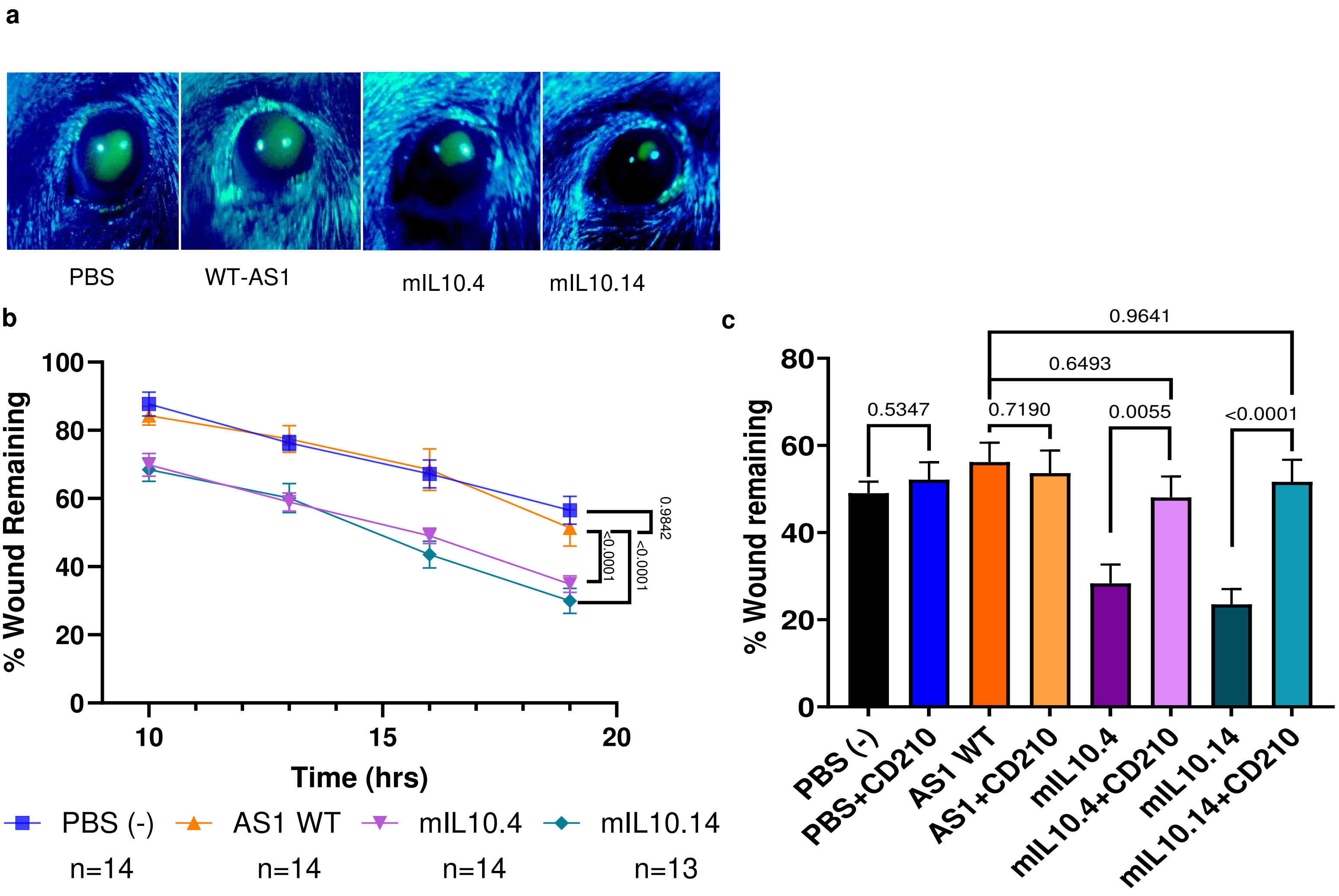

**Figure 4. IL-10 produced by *C. mast* can speed wound healing in an IL-10 dependent manner.** (a-c) Mouse eyes were inoculated with 5x10[6] CFU of mutant or WT bacteria once every other day for three inoculations. One week after the final inoculation, a 2.5mm abrasion of the corneal epithelium was made using an Alger brush. Fluorescein was used at the time of injury, then at 10 hours and every 3 hours after to measure the area of unhealed epithelium of each mouse. (a) Representative images of unhealed area of corneas, at 19 hours after wounding**.** (b) Percent wound remaining was determined by measuring areas of corneal wounds using FIJI at each time point, and this area was compared to the T0 values. Bars represent the mean percentage of wound remaining ± SEM. Data are pooled from three independent experiments. Area under each curve was calculated and the mead and SD was evaluated for significance using ANOVA (F=51.99) (c) Twenty-four hours prior to corneal wound, mice were given in i.p. injection of aCD210 to neutralize the IL-10R. Percent wound remaining was calculated at T=19 hours. Bars represent the mean % wound remaining ± SEM. Data were pooled from three independent experiments and statistical significance across all samples was determined using ANOVA (n=13-15, F=8.106, df=(7,103)). Differences between samples treated and untreated with CD210 were analyzed using an unpaired t-test(PBS(t=0.6295, df=25);AS1(t=0.3637, df=26); 4(t=3.035, df=25);14(t=4.613, df=27)).

**Table1. Primers used in this study**

| Primer Name | Sequence | Use |
|---|---|---|
| PhoZ NcoI F | tacactCCATGGactgTTAATTAAatctgaacagaaaagc | amplification of PhoZ from E.feacalis |
| PhoZ NotI R | tacactGCGGCCGCcgatGACGTCttatttaccaat | amplification of PhoZ from E.feacalis |
| Kanp Nco F | ATCTCCATGGtaagataatatatcttttatatagaagatatcgc | amplification of Kanamycin resistance gene |
| Kanp Mfe R | ccacCAATTGattTCCTTCCTCTTttc | amplification of Kanamycin resistance gene |
| PhoZ Kan Homo R | cataaagcttgctcaatcaatcaccGACGTCttatttaccaatacctttatctttaatc | homologous overlap of Kanamycin resistance gene cassette for OEP |
| Kan PhoZ homo F | gattaaagataaaggtattggtaaataaGACGTCggtgattgattgagcaagctttatg | homologous overlap of PhoZ gene cassette for OEP |
| PhoZ TN5 F | P'CTGTCTCTTATACACATCTTTAATTAAatctgaacagaaaagc | amplification of phosphorylated PhoZ transposon with mosiac end |
| Kan Prom TN5 F | P'CTGTCTCTTATACACATCTCCATGGtaagataatatatc | amplification of phosphorylated PhoZ transposon with mosiac end |
| Ermx IL10 homo F | ccagtagtactaaaagggctcGACGTCGCGGCCGCtta | homologous overlap of IL10 gene cassette for OEP |
| Il10 Ermx homo R | taaGCGGCCGCGACGTCgagcccttttagtactactgg | homologous overlap of Erythromycin resistance gene cassette for OEP |
| TN5 21 IL-10 F | P'CTGTCTCTTATACACATCTCCATGGtaaatagagatctacagtatatggctaaatg | amplification of phosphorylated IL-10 transposon with mosiac end |
| Ermx TN5 | P'CTGTCTCTTATACACATCTcggcggtggcatcattct | amplification of phosphorylated IL-10 transposon with mosiac end |

## Supplementary Files

This is a list of supplementary files associated with this preprint. Click to download.

- Sourcedatafile.xlsx
